# Estrogen receptor, progesterone receptor, interleukin-6 and interleukin-8 are variable in breast cancer and benign stem/progenitor cell populations

**DOI:** 10.1186/1471-2407-14-733

**Published:** 2014-09-30

**Authors:** Robynn V Schillace, Amy M Skinner, Rodney F Pommier, Steven O’Neill, Patrick J Muller, Arpana M Naik, Juliana E Hansen, SuEllen J Pommier

**Affiliations:** Division of Surgical Oncology, Department of Surgery, Oregon Health & Science University, Mail Code L619, 3181 SW Sam Jackson Park Rd, Portland, OR 97239-3011 USA; Division of Plastic Surgery, Department of Surgery, Oregon Health & Science University, Portland, USA

**Keywords:** Breast cancer, Stem cell, Estrogen receptor, Progesterone receptor, Interleukin-6, Interleukin-8, Proximity ligation assay, Protein

## Abstract

**Background:**

Estrogen receptor positive breast cancers have high recurrence rates despite tamoxifen therapy. Breast cancer stem/progenitor cells (BCSCs) initiate tumors, but expression of estrogen (ER) or progesterone receptors (PR) and response to tamoxifen is unknown. Interleukin-6 (IL-6) and interleukin-8 (IL-8) may influence tumor response to therapy but expression in BCSCs is also unknown.

**Methods:**

BCSCs were isolated from breast cancer and benign surgical specimens based on CD49f/CD24 markers. CD44 was measured. Gene and protein expression of ER alpha, ER beta, PR, IL-6 and IL-8 were measured by proximity ligation assay and qRT-PCR.

**Results:**

Gene expression was highly variable between patients. On average, BCSCs expressed 10-10^6^ fold less ERα mRNA and 10-10^3^ fold more ERβ than tumors or benign stem/progenitor cells (SC). BCSC lin-CD49f^−^CD24^−^cells were the exception and expressed higher ERα mRNA. PR mRNA in BCSCs averaged 10-10^4^ fold less than in tumors or benign tissue, but was similar to benign SCs. ERα and PR protein detection in BCSCs was lower than ER positive and similar to ER negative tumors. IL-8 mRNA was 10-10^4^ higher than tumor and 10^2^ fold higher than benign tissue. IL-6 mRNA levels were equivalent to benign and only higher than tumor in lin-CD49f^−^CD24^−^cells. IL-6 and IL-8 proteins showed overlapping levels of expressions among various tissues and cell populations.

**Conclusions:**

BCSCs and SCs demonstrate patient-specific variability of gene/protein expression. BCSC gene/protein expression may vary from that of other tumor cells, suggesting a mechanism by which hormone refractory disease may occur.

**Electronic supplementary material:**

The online version of this article (doi:10.1186/1471-2407-14-733) contains supplementary material, which is available to authorized users.

## Background

Breast cancer treatment options are based partially upon immunohistochemical staining of tissue specimens for the expression of hormone receptors. Expression of estrogen and progesterone receptors leads to specific therapeutic strategies, including tamoxifen and aromatase inhibitors. These strategies have been followed for decades. The data at a 15 year endpoint indicate that 5 years of tamoxifen therapy will reduce the disease recurrence rate 11.8% and the mortality rate 9.8% [1]. These data are encouraging and support continued use of traditional tamoxifen therapy, but the fact that approximately 30% of patients still relapse indicates research to improve outcomes is warranted.

One hypothesis as to why disease recurs in the presence of tamoxifen therapy is that the bulk of the estrogen receptor positive tumor cells are destroyed by treatment, but tumor initiating cells that are negative for estrogen receptor expression persist. Tumor initiating cells, or cancer stem cells, represent a small percentage of cells that make up breast tumors but have the ability to induce growing tumors in immunodeficient mice [2]. Al-Hajj and colleagues demonstrated that as few as 1000 CD44^high^/CD24^low^ cells isolated from human breast cancer could develop a tumor in immunodeficient mice [3]. However, CD44^high^/CD24^low^ cells may not be the universal breast cancer stem cell profile, as mammospheres from a pleural effusion lacking CD44^high^/CD24^low^ cells, and CD49f^low^/CD24^high^ cells from the infiltrating ductal carcinoma cell line (HCC 1954) could also generate tumors in immunodeficient mice [4, 5]. Furthermore, the CD44^high^/CD24^low^ cancer stem cell phenotype was shown to be similar to the bipotent progenitor cell phenotype CD49f^high^/MUC1^neg^, with CD44 and CD49f being widely distributed among mammary epithelial cells and expressed by both luminal restricted and bipotent progenitors [6]. Thus, data generated using CD44^high^/C24^low^ and CD49f^low^/CD24^high^ sorted cell populations suggest that mammary repopulating units and/or bipotent progenitor cells may be functioning as cancer stem cells in tumors.

Recent studies suggest that measuring estrogen receptor (ER) and progesterone receptor (PR) gene expression in individual intra- and extra- tumoral cells generates additional clinically relevant information. Aktas and colleagues demonstrated that in 77% of their patients with ER positive tumors (ER^pos^), circulating tumor cells were negative for ER gene expression [7]. Heterogeneity of hormone expression is well documented in breast cancers [8] but a detailed correlation of the receptor status of tumor cell subpopulations and clinical impact has yet to be completed. Studies suggest that ER gene expression is low in human CD44/CD24 [9] and mouse CD49f/CD24 [10] sorted cell populations.

Protein expression of ER and PR in tumor samples was historically measured using ligand binding assays [11, 12]. The development of monoclonal antibodies led to utilization of enzyme immunoassays [13]. Advancements in embedding, sectioning and antigen retrieval in tumor specimens contributed to immunohistochemistry becoming the current standard for clinical evaluation of biopsy and tumor specimens [14]. These methods measure ER or PR in whole fixed tumor samples and thereby prohibit the study of live cells. The study presented herein, in contrast, is the first to measure the gene and protein expression of ER and PR in uncultured CD49f/CD24 stem and progenitor sorted cell populations (BCSCs) from freshly isolated benign breast tissue or human invasive ductal carcinomas. The proximity ligation assay for detecting protein expression has been used for years [15, 16], but this study represents the first use of this technology in breast cancer stem/progenitor cells.

A growing body of research indicates that pro-inflammatory cytokines can facilitate tumor growth and metastasis [17, 18]. Interleukin-6 (IL-6) is a key factor in regulating estrogen activity through stimulation of aromatase, steroid sulphatase and 17β-hydroxysteroid dehydrogenase [19, 20]. Studies have also demonstrated a positive correlation between IL-6 and ERα expression in breast tumors in a manner thought to be stem cell mediated [21, 22]. In contrast, Interleukin-8 (IL-8) was shown to have an inverse correlation with ERα expression in breast tumors, and IL-8 increases the invasive potential of breast cancer cells [23, 24]. These data suggest that IL-6 and IL-8 pro-inflammatory cytokines may affect tamoxifen response or aromatase inhibition through modulation of hormone activity. Thus, to further delineate the role that stem cells may play in tumor progression through the evasion of hormone-based therapies, IL-6 and IL- 8 gene and protein expression were measured and correlated with ER and PR expression in BCSC.

## Methods

### Benign and malignant tissue procurement and cell culture

This study was approved by the Oregon Health & Science University institutional review board. Benign and malignant specimens, clinical data and consent to publish clinical details from patients included in this study, were obtained with informed written consent in accordance with an IRB approved protocol. Twenty-nine invasive ductal carcinomas were obtained at the time of mastectomy or lumpectomy prior to neoadjuvant treatment. Thirteen pathologically confirmed benign breast tissue specimens were obtained from reduction mammoplasty. ER and PR tumor status were obtained from pathological evaluation of biopsy specimens according to ASCO guidelines [14]. MCF10A (ATCC, CRL-10317) and breast cancer cell lines, MCF7 (ATCC, HTB-22), T47D (ATCC, HTB-133) and HCC1806 (ATCC, CRL-2335) were authenticated by ATCC and confirmed through morphological examination and growth curve analysis. Cell lines were maintained as recommended by ATCC.

### Collection of breast cancer stem/progenitor cells (BCSCs)

All specimens were minced and digested in mammary epithelial cell-specific medium containing 1× collagenase/hyaluronidase (Epicult, StemCell Technologies). Cell lines were cultured in Roswell Park Memorial Institute (RPMI)-1640 supplemented with 10% serum and 0.05% Gentamicin. Approximately 10^6^ cells were labeled with monoclonal antibodies against human CD45-FITC, CD31-FITC, CD24-PE, CD49f-PE-Cy 5, and CD44-PE-Cy7. Isotype control testing excluded nonspecific binding. Surface antibody labeling and collection by discriminatory gating were used to remove CD31^+^/CD45^+^ endothelial cells and leukocytes (lineage negative; lin^neg^) and to collect four lin^neg^ populations of benign and malignant SCs: CD49f^+^CD24^+^ (PP), CD49f^+^CD24^−^(PM), CD49f^−^CD24^+^(MP), and CD49f^−^CD24^−^(MM). CD44 expression was measured.

### PCR amplification of genetic material

Gene expression in BCSCs and benign stem cells (SCs) was determined by quantitative real-time PCR using Taqman low density array (TLDA) technology (Life technologies, Carlsbad, CA). RNA was isolated using the Qiagen Mini RNeasy kit (Qiagen, Valencia, CA). cDNA was produced using random hexamers (Superscript III First Strand Kit, Invitrogen). An average of 50 ng of cDNA, 15 μl TaqMan’s PreAmp Master Mix (2×) (Applied Biosystems) and 7.5 μl of TaqMan custom PreAmp Pool (Applied Biosystems) were combined. cDNA was amplified for 14 cycles (95°C 10 min, 95°C 15 s, 60°C 4 min). Pre-amplified cDNA was utilized as per manufacturer’s protocol using custom TLDA cards on the Viia7 Real-Time PCR system. Data were included in the analyses if the endogenous control 18S rRNA had a C_t_ value of 28 or less and triplicate values were within 0.5 C_t_ of each other. Delta C_t_ (dC_t_) values were calculated by subtracting the 18S rRNA C_t_ value from the target C_t_ value. Thus, dC_t_ values are inversely related to gene expression (i.e. negative dC_t_ values indicate high levels of gene expression).

### Preparation of protein lysates and the proximity ligation assay (PLA)

Given the rarity of BCSCs and the small size of some breast cancers, traditional western blot analysis of protein expression was not possible in this study. As an alternative approach, proximity-dependent DNA ligation assays (PLA) were utilized to detect protein expression [15, 16]. PLAs were conducted according to manufacturer’s protocol (PLA, Life Technologies, Carlsbad, CA) with the following modifications. Approximately 50,000 cells were lysed in 100 μl total volume and serially diluted. For sorted cell populations with less than 50,000 cells available, lysis volume was reduced to 50 μl. Samples were run in triplicate. IL-6 and IL-8 antibody probes (IL-6 BAF206; IL-8 BAF391, biotinylated polyclonal goat, R&D Systems) were made as per manufacturer’s protocol (Life Technologies). ERα and PR antibodies (ERα, AF5715; PR-AF5415; sheep polyclonal, R&D Systems, Minneapolis, MN) were biotinylated using Biotin-XX Microscale Protein labeling kit (B30010, Life Technologies). ERβ antibody (S2015, polyclonal rabbit, Epitomics, Burlingame, CA) was desalted before biotinylation. Amplification was performed (ABI Viia7 RT-PCR system), and dC_t_ values were calculated by subtracting the sample C_t_ from the no protein control C_t_. In contrast to gene expression analyses, a positive dC_t_ value correlates with an increase in protein detection over background.

### Statistics

Statistical analyses were conducted in the form of two-tailed Student’s t-Test with p ≤ 0.05 values considered significant. Pairing was utilized when comparing sorted cells to the tissue or tumor of origin. Unpaired analyses with unequal variance were performed when comparing tumors or tumor sorted cells to benign tissues or benign sorted cells.

## Results

### Estrogen receptor gene expression in tumors correlated with pathological IHC analyses

**Figure 1:**
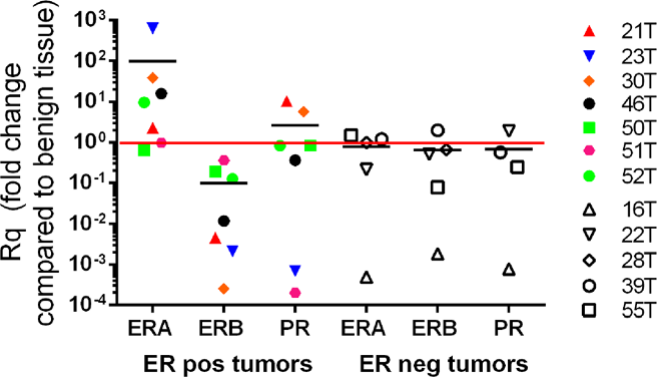
**ERA, ERB and PR detection in invasive ductal carcinoma.** Rq (fold change) is the comparison of the expression in each individual tumor to the average of 7 benign tissues. Black bars indicate median values. ER positive (ER pos), or ER negative (ER neg) refers to IHC characterization by pathology. Delta C_t_ (dC_t_) values were calculated by subtracting the 18S rRNA C_t_ value from the target C_t_ value. Thus, dC_t_ values are inversely related to gene expression (i.e. negative dC_t_ values indicate high levels of gene expression).

Table 1 lists the ER and PR status of the breast cancers included in this study. Tumor hormone status was determined as part of the routine diagnostic testing for all breast tumor biopsies by immunohistochemical (IHC) staining of paraffin-embedded tissue samples as per ASCO guidelines (14). Estrogen receptor alpha (ERA) mRNA was measured in tumors for which a pathologic ER status was known. In tumors determined ER positive by IHC (ER^pos^), detection of ERA expression was ten-fold higher than in benign tissue (Figure 1). Moreover, detection of estrogen receptor beta (ERB) mRNA was more than 10-fold less in ER^pos^ tumors than benign tissue. ER negative (ER^neg^) tumors exhibited similar levels of ERA and ERB compared to benign tissue.

**Table 1:** **Age and hormone status as determined by OHSU pathology**

Tumor samples	Age	ER status by IHC	PR status by IHC
21 T	40	Positive	Positive
23 T	67	Positive	Positive 60%
30 T	45	Positive	Positive
46 T	64	90%	Positive 35%
50 T	79	Positive	Positive
51 T	60	Positive	Positive 3%
52 T	58	Positive	Positive
53 T	60	Positive	Positive 80%
54 T	51	Positive	Positive
13 T	52	Positive	Positive 95%
19 T	72	Positive	Positive 95%
20 T	60	Positive	Positive 95%
78 T	79	Positive	Positive 30%
79 T	59	Positive	Positive 75%
82 T	90	Positive	Positive
85 T	61	Positive	Positive 60%
88 T	59	Positive	Negative
103 T	78	93%	Positive 26%
113 T	54	Positive	Positive 80%
115 T	67	Positive	Positive 100%
16 T	59	Negative	Negative
22 T	73	Negative	Negative
28 T	59	Negative	Negative
39 T	52	Negative	5%
55 T	54	Negative	Negative
69 T	46	Negative	Negative
102 T	69	Negative	Negative
112 T	35	Negative	Negative
122 T	54	Negative	Negative

Positive denotes samples that are greater than 95% positive unless otherwise indicated. Median age of patients with ER positive tumors is 60 ± 12.7 yr. (range 40–90 yr., mode = 60 yr.). Median age of patients with ER negative tumors is 54 ± 12.1 yr. (range 35–73 yr., mode = 59 yr.).

### CD44 expression is highest in CD49^+^CD24^+^ cells

**Figure 2:**
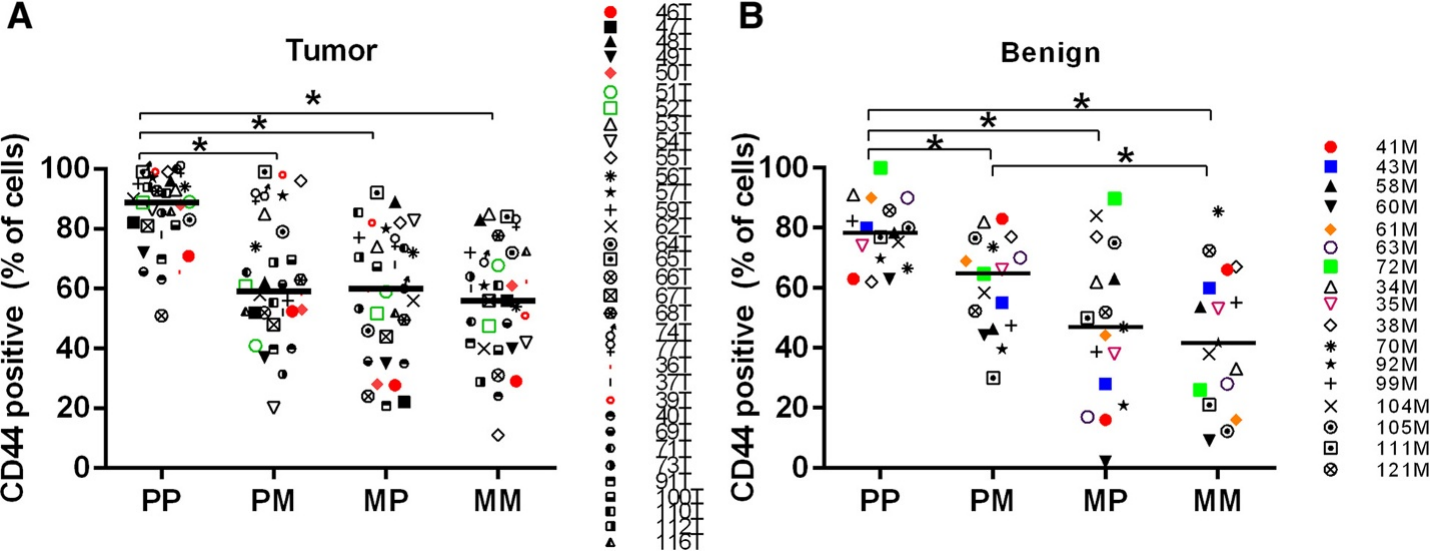
**CD44 expression on CD49f/CD24 sorted cells.** CD49f + CD24+ (PP), CD49f + CD24- (PM), CD49f-CD24+ (MP), and CD49f-CD24- (MM). **A)** Tumor samples, **B)** Benign Samples Analysis to determine% of positive cells was performed using FlowJo® Software. Each symbol represents a unique specimen. Black line indicates median values. (*) indicates p ≤ 0.05 for each bracket.

Four lin^neg^ cell populations were collected from each benign tissue or tumor sample. The lin^neg^ sorted cell populations were CD49f^+^CD24^+^(PP), CD49f^+^CD24^−^(PM), CD49f^−^CD24^+^(MP), and CD49f^−^CD24^−^(MM). Measurement of CD44 expression indicated that in 80% of tumors, CD49f^+^CD24^+^(PP) cell populations were greater than 75% CD44 positive (51-99%, Figure 2A); in contrast, CD49f^+^CD24^−^(PM), CD49f^−^CD24^+^(MP), and CD49f^−^CD24^−^(MM) cell populations exhibited a range of CD44 expression: PM (range: 11-84%), MP (range: 20-92%), and MM (range: 11-84%). Even with this range of expression, CD44 levels detected in these three BCSC populations were significantly lower than CD44 levels in CD49f^+^CD24^+^(PP) cell populations. Benign CD49f^+^CD24^+^(PP) cells were significantly less positive for CD44 expression than BCSCs (62-100% p = 0.036, Figure 2B). CD44 levels in benign CD49f^+^CD24^−^(PM), CD49f^−^CD24^+^(MP), and CD49f^−^CD24^−^(MM) cells also exhibited a range of CD44 expression: PM (range: 30-83%), MP (range: 2-89%) and MM (range: 9-85%), but again were significantly lower than CD44 levels in benign CD49f^+^CD24^+^(PP) cells.

### Estrogen receptor gene expression was variable in human BCSCs, but highest in CD49f^−^CD24^−^ cells

**Figure 3:**
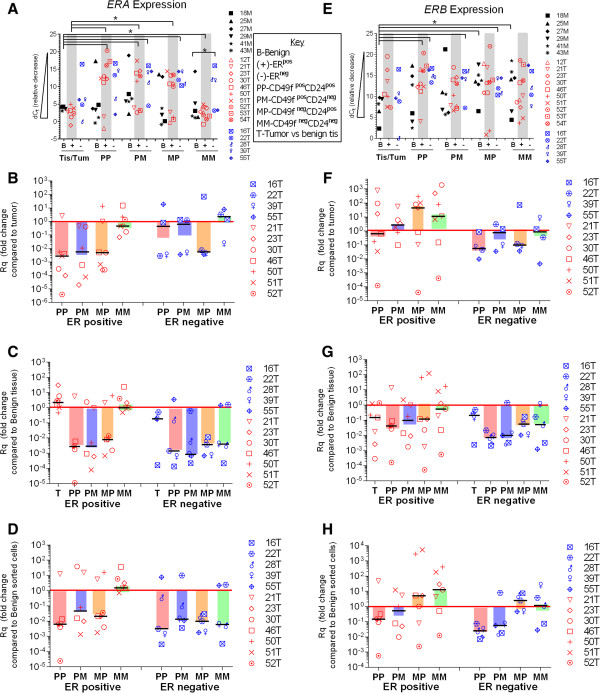
**ERA and ERB detection in CD49f/CD24 sorted cell populations. A-D)** ERA expression, **E-H)** ERB expression. **A, B)** dC_t_ values were obtained by subtracting 18S rRNA from gene of interest. dC_t_ values are inversely proportional to expression. Black symbols: benign tissue samples **(B)**, red symbols: IHC designated ER positive IDC tumor samples (+), blue symbols: IHC designated ER negative IDC tumor samples (−). In **B**-**D** and **F**-**H** Bars indicate values obtained when Fold change (RQ) values were calculated from averaged samples. Symbols indicate fold changes for individual data points. Black lines indicate median values. **B, F)** Fold change (RQ) when sorted cell values were compared to tumor of origin. **C-G)** Fold change (RQ) when sorted cell values from tumors were compared to averaged benign tissue values. **D**-**H**) Fold change (RQ) when sorted cell values from tumors were compared to averaged sorted cell values from benign tissue. (*, p-value <0.05 for each bracket).

Detection of ERA and ERB mRNA in sorted cell populations isolated from ER^pos^ tumors, ER^neg^ tumors and benign tissues is presented in Figure 3. The delta C_t_ (dC_t_) data, which are inversely correlated with expression, indicate that ERA and ERB expression levels in BCSCs and benign SCs were highly variable between patient samples (dC_t_ range: −4 to 20, Figure 3A, B). dC_t_ data were analyzed to generate fold change (Rq) comparisons between BCSCs and the tumor of origin. In ER^pos^ tumors, 70% (17/22) of BCSCs expressed 10-10^6^ fold less ERA than tumor of origin (Figure 3B), while 57% of BCSCs expressed 10-10^3^ fold more ERB than tumor of origin (Figure 3F). In ER^neg^ tumors 50% of BCSCs expressed 10^3^ fold less ERA and ERB than tumor of origin (Figure 3B, F). When compared to benign tissue or benign SCs, ERA expression was 10-10^5^ fold lower in 72% of BCSCs from ER^pos^ tumors and 10-10^4^ fold lower in 85% of BSCSs from ER^neg^ tumors (Figure 3C, D). Seventy-three percent of BCSCs from ER^pos^ and 85% from ER^neg^ tumors, expressed 10-10^5^ fold less ERB than benign tissue (Figure 3G). But when compared to benign SCs, BCSC ERB expression was higher in 50% of ER^pos^ and 30% of ER^neg^ tumors (Figure 3H). Of note, the CD49f^neg^ populations were the exception in which detection of ERA was higher in CD49f^−^CD24^−^(MM) BCSC than tumor regardless of tumor status (Figure 3B), and detection of ERB was higher in both CD49f^−^CD24^+^(MP) and CD49f^−^CD24^−^(MM) populations compared to tumor of origin and compared to benign SCs (Figure 3F and H).

### PR gene expression did not correlate with ER expression

**Figure 4:**
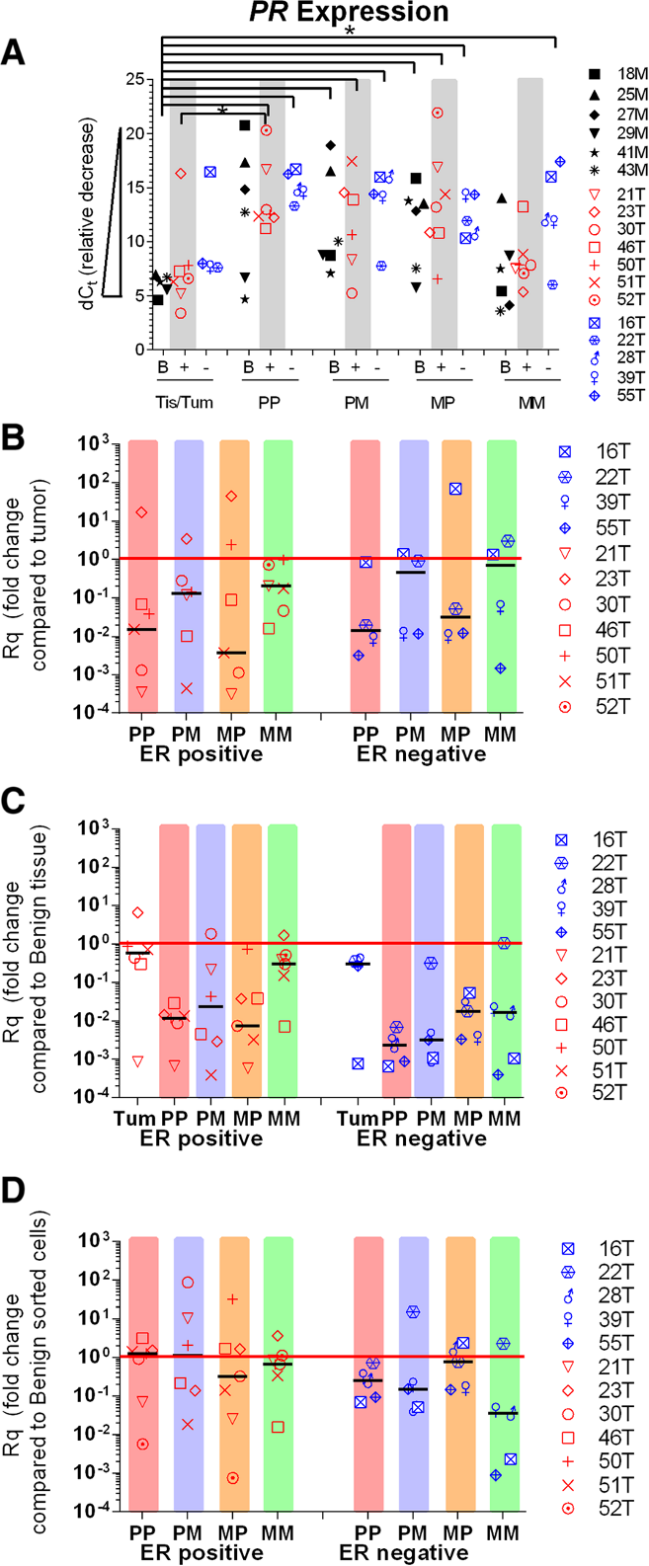
**PR detection in CD49f/CD24 sorted cell populations. A)** dC_t_ values obtained by subtracting 18S rRNA from gene of interest. dC_t_ values are inversely proportional to expression. Black symbols: benign tissue samples **(B)**, red symbols: IHC designated ER positive IDC tumor samples (+), blue symbols: IHC designated ER negative IDC tumor samples (−). **B)** Fold change when sorted cell values were compared to tumor of origin. **C)** Fold change when tumor sorted cell values were compared to averaged benign tissue values. **D)** Fold change when sorted cell values from tumor samples were compared to averaged sorted cell values from benign tissue. Black bars indicate median values. (*, p-value <0.05).

PR gene expression levels in BCSCs, benign SCs and tumor or tissue of origin are shown in Figure 4. Estrogen is a transcriptional activator of progesterone receptor (PR) [25]; therefore, the presence of functional ER protein is expected to correlate with increased levels of PR message. In this study, PR expression was generally similar between benign tissue and tumors regardless of ER status. Detection of PR was significantly higher in benign tissue than in seven BCSC and two benign SC populations (Figure 4A). PR in 85% of BCSCs from ER^pos^ tumors was 10–10,000 fold less and PR in 62% of BCSCs from ER^neg^ tumors was about 100 fold less than in tumor of origin (Figure 4B). Detection of PR in 90% of BCSCs from ER^pos^ and ER^neg^ tumors was 10–10,000 fold lower than in benign tissue (Figure 4C). Comparison of PR expression between BCSCs and benign SC reveals more similarly in levels of expression than those seen for ERA (Figure 4D).

### IL-6 and IL-8 genes were differentially expressed in BCSCs

**Figure 5:**
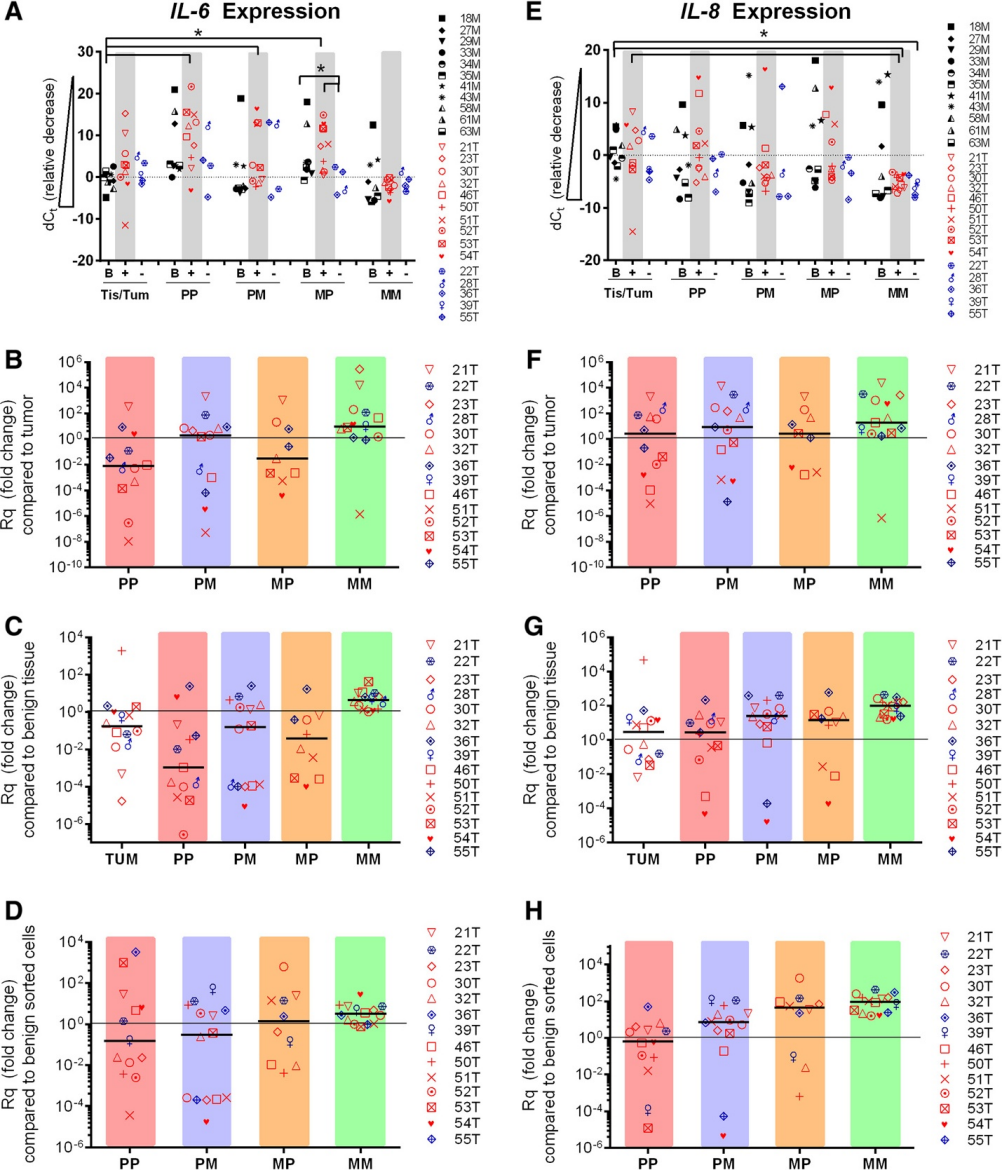
**IL-6 and IL-8 expression in CD49f/CD24 sorted cell populations. A** **and** **E)** dC_t_ values obtained by subtracting 18S rRNA from gene of interest. dC_t_ values are inversely proportional to expression. Black symbols: benign tissue samples **(B)**, red symbols: IHC designated ER positive IDC tumor samples (+), blue symbols: IHC designated ER negative IDC tumor samples (−). Black bars indicate median values. **B** (IL-6) and **F** (IL-8) Fold change when sorted cell values were compared to tumor of origin. **C** (IL-6) and **G** (IL-8) Fold change when tumor sorted cell values were compared to averaged benign tissue values. **D** (IL-6) and **H** (IL-8) Fold change when sorted cell values from tumor samples were compared to averaged sorted cell values from benign tissue. (*, p-value <0.05).

Finally, because the presence of IL-6 and IL-8 in tumor cells may be surrogate markers for ER activation [21, 23], IL-6 and IL-8 mRNA levels (IL-6, IL-8) were examined (Figure 5). Experiments reveal that IL-6 expression was comparable with 18S rRNA in benign tissues and ER^neg^ tumors (Figure 5A). ER^pos^ tumors exhibited a range of IL-6 expression (dC_t_ -2 to 22) that was usually lower than 18S (Figure 5A). When compared to tumor of origin IL-6 expression was significantly elevated (10-10^6^ fold) in the CD49f^−^CD24^−^(MM) population and a majority of CD49f^+^CD24^−^(PM) populations, while significantly decreased an average 100 fold in the CD49f^+^CD24^+^(PP) populations when compared to tumor (Figure 5B). When compared to benign tissue, IL-6 expression was 10 fold greater in the CD49f^−^CD24^−^(MM) population, but significantly lower in the CD49f^−^CD24^+^(MP) (1-10^4^ fold) and CD49f^+^CD24^+^(PP) populations (5-10^6^ fold) (Figure 5C). Interestingly, a bimodal expression pattern was observed in the CD49f^+^CD24^−^(PM) population, with six specimens exhibiting about 10 fold increased expression and six specimens exhibiting 10^4^ fold decreased expression compared to benign tissue (Figure 5C). When BCSCs were compared benign SCs, IL-6 was elevated 20 fold in the CD49f^−^CD24^−^(MM) population, but a range of expression was detected in CD49f^+^CD24^+^ (PP) (10^4^ to 10^−4^ fold) and CD49f^−^CD24^+^(MP) (10^2.5^ to 10^−2^ fold) populations. A bimodal pattern of expression was again observed in the CD49f^+^CD24^−^(PM) populations (20 fold vs. 10^−4^ fold) (Figure 5D).

IL-8 expression was variable in benign tissue and tumor samples (dC_t_ range: −10 to 20) (Figure 5E). On average, more IL-8 was detected in sorted cells than in whole tumor or tissue. IL-8 expression in benign SCs and BCSCs from ER^pos^ tumors was variable while BCSCs from ER^neg^ tumors exhibited consistently higher levels of IL-8 mRNA than 18S. Fold change analyses revealed significantly elevated levels of mRNA expression when compared to tumor of origin in the CD49f^−^CD24^−^(MM) population (10-10^5^ fold increases). Compared to tumor, IL-8 levels were on average 10 fold higher in CD49f^+^CD24^−^(PM) cells and 5 fold higher in CD49f^−^CD24^+^(MP) cells. CD49f^+^CD24^+^(PP) cells exhibited highly variable (10^−5^ - 10^4^) IL-8 expression (Figure 5F). IL-8 expression was 20–100 fold higher in BCSCs than in benign tissue for most samples (CD49f^−^CD24^−^(MM) population, p < 0.05) (Figure 5G). Finally, when BCSCs were compared to benign SCs, a 100 fold increase in IL-8 expression in the CD49f^−^CD24^−^(MM) and CD49f^−^CD24^+^(MP) populations, a 20 fold increase in CD49f^+^CD24^−^(PM) cells, and a broader range of expression in the CD49f^+^CD24^+^(PP) population (10^−2^-10^2^) were observed (Figure 5H).

### Protein expression was determined by proximity ligation assay (PLA)

**Figure 6:**
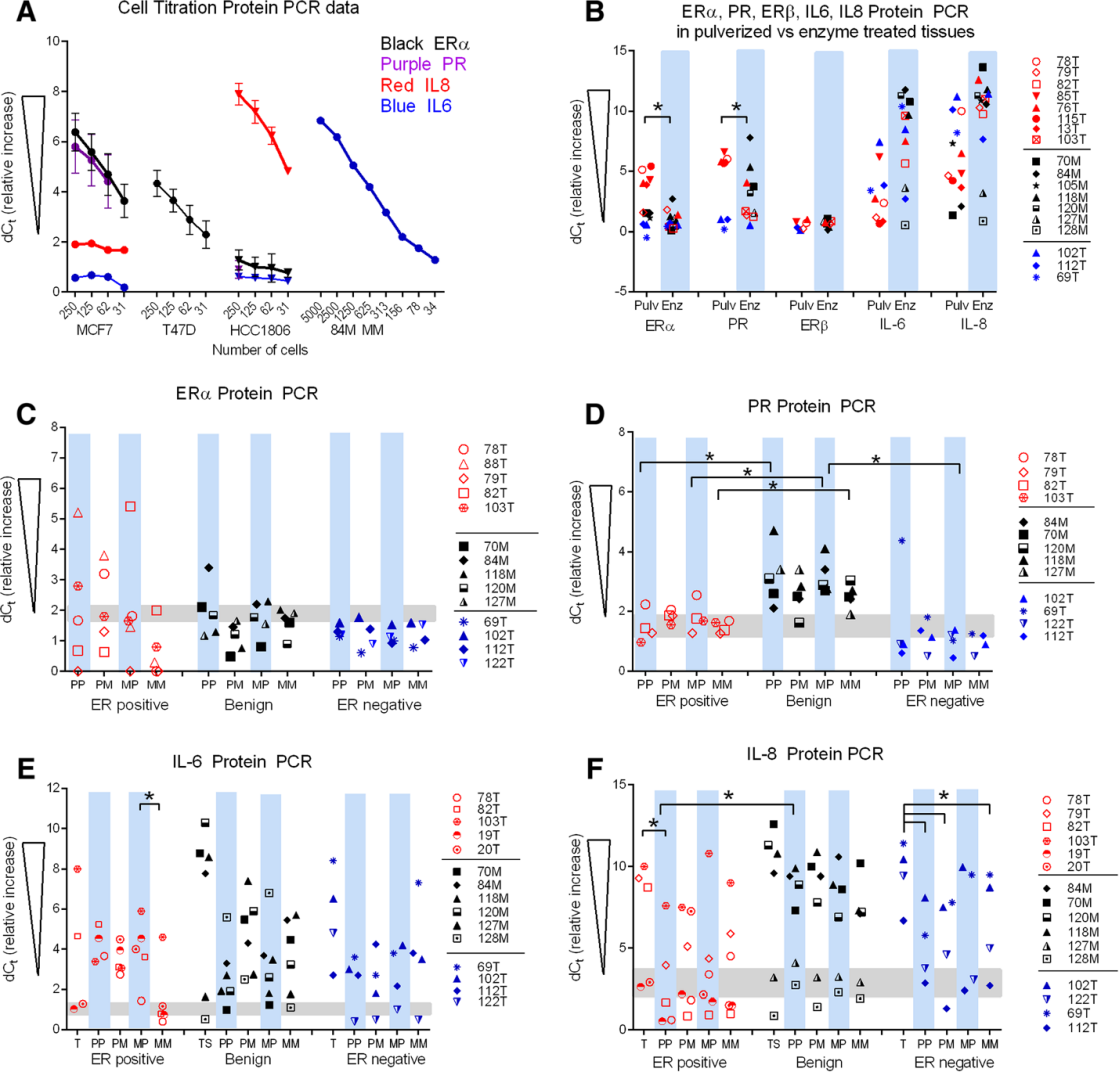
**ERα, PR, IL-6 and IL-8 protein expression determined by proximity ligation assay (RT-Protein PCR).** dC_t_ values were obtained by subtracting sample C_t_ values from no protein control values. In contrast to gene expression, for protein PCR results, increases in dC_t_ values correlate with increased protein expression. **A)** Detection of proteins in ER^pos^ MCF7 and T47D cells and ER^neg^ HCC 1806 cells at titrations of 250, 125, 62, 31 cells. Benign sample 84 M was used to determine titration for detecting IL-6 protein (cell titration 5000, 2500, 1250, 625, 313, 156, 78, 34 cells). Black symbols: benign tissue samples, red symbols: IHC designated ER positive IDC tumor samples, blue symbols: IHC designated ER negative IDC tumor samples. **B)** Protein detection in pulverized (Pulv) versus enzyme treated (Enz) tumor or tissue lysates. **C-F)** Protein detection normalized to 250 cells, **C)** ERα, D) PR, E) IL-6, and **F)** IL-8. Grey box indicates values considered equivocal. * indicates p < 0.05, for populations compared by bracket.

Protein expression was determined for ER, PR, IL-6 and IL-8 in freshly isolated BCSCs and benign SC (Figure 6) and compared to gene expression data. Breast cancer cell lines MCF7 and T47D were used as positive controls for ERα, ERβ, and PR, and as a negative control for IL-8^neg^ (Figure 6A). HCC1806 cells served as a negative control for ERα, ERβ, and PR and as a positive control for IL-8. IL-6 was not detected in MCF7, T47D or HCC1806, but was expressed in a tissue control (84 M). ERα, ERβ, PR, IL-6 and IL-8 expression were determined by serial titration of cell lysates followed by PLA. Titrations of cell lysates established the relationship between cell number and protein detection. Sensitivity of detection was determined for each probe. The region in between the lowest positive value detected and the highest level of background detected was defined as equivocal. This area is indicated by a grey box in Figure 6C-F. Values above the box were considered positive for expression while values below the box were considered negative. In agreement with western blot studies [26], PLA indicated that MCF7 and T47D expressed ERα, and that MCF7 cells had higher amounts of ERα than T47D cells (Figure 6A).

### The method of cell isolation influences PLA results

In contrast to expectations, levels of ERα protein in ER^pos^ tumor lysates and unsorted benign tissue lysates were both comparable to no-protein controls when measured following overnight digestion with collagenase/hyaluronidase ([27, 28] (Figure 6B). Detection of IL-6 and IL-8 in these enzyme treated lysates provided evidence that these samples as a whole are not degraded. When lysates were made from tissue that had been pulverized in liquid nitrogen, higher levels of detection of ERα and PR protein were achieved, i.e. levels comparable with those found in MCF7 lysates (Figure 6B). Detection of ERα and PR were not above background in ER^neg^ tumor lysates whether pulverized or enzyme treated. Interestingly, IL-6 and IL-8 levels were lower in pulverized lysates than in enzyme treated lysates. The data further indicate that the method of lysate preparation may influence the results obtained.

### ERα, ERβ and PR protein expression in tumors, benign tissue, SCs and BCSCs

Overall, ERα levels were not significantly different between BCSCs obtained from ER^pos^, ER^neg^ tumors or benign tissues. However, among BCSCs obtained from ER^pos^ tumors, ERα levels varied from background levels (dCt ≈ 1.0) to levels similar to those detected in ER^pos^ tumors and MCF7 cells (dCt ≈ 3.0-6.0) (Figure 6C). ERα levels in benign SCs were less than benign tissues. The ERα levels in BCSCs from ER^neg^ tumors were comparable to or less than expression detected in ER^neg^ tumors and HCC1806 cell line.

Two different sets of antibodies were used as probes to detect ERβ protein (see Methods), but neither set successfully detected ERβ in the positive control MCF7 nor T47D cells [29–31] (Additional file 1: Figure S1A). In whole tumor or tissue lysates, detection of ERβ was comparable to or less than that found in HCC1806. Given the lack of validation of these probes in positive cell lines, ERβ protein levels could not be completely quantified when measured by PLA. However, detection of ERβ was above no protein control background levels in BCSC and benign SC. Also, BCSCs and benign SCs contained higher levels of ERβ than whole tumor or benign tissue (Additional file 1: Figure S1B).

Significantly higher levels of PR protein were found in benign SCs compared to BCSCs (Figure 6D). Specifically, benign CD49f^+^CD24^+^(PP), CD49f^−^CD24^+^(MP), and CD49f^−^CD24^−^(MM) populations were significantly higher than their ER^pos^ BCSC counterparts, and benign CD49f^−^CD24^+^(MP) cells expressed significantly more PR than ER^neg^ CD49f^−^CD24^+^(MP) cells. While there were no significant differences in PR expression between ER^pos^ and ER^neg^ BCSCs, more PR protein was detected in ER^pos^ than ER^neg^ tumors.

### IL-6 and IL-8 cytokine protein expression in tumors, benign tissue, SCs and BCSCs

IL-6 levels were also high in benign tissue and SCs, but in contrast to PR, detection of IL-6 was higher in CD24^neg^ cells rather than in CD24^pos^cells (Figure 6E). IL-6 protein in ER^pos^ CD49f^+^CD24^+^(PP) cells was slightly higher than in CD49f^+^CD24^−^(PM) cells while expression in CD49f^−^CD24^+^(MP) cells was significantly higher than that in CD49f^−^CD24^−^(MM) cells. ER^neg^ BCSCs also expressed less IL-6 than ER^neg^ tumor, but similarity of expression between 112 T tumor and BCSCs precluded significance. Published studies suggest that IL-6 expression correlates with ER expression [21]. However, in our gene expression studies more IL-6 was detected in ER^neg^ tumors than in ER^pos^ tumors, and protein expression was similar in ER^pos^ and ER^neg^ BCSCs (Figures 5 and Figure 6E).

Akin to IL-6 and PR, the highest levels of IL-8 protein were consistently detected in benign tissue and SCs, but comparable levels were also found in some ER^neg^ tumors and corresponding BCSCs (Figure 6F). These levels were similar to or greater than those detected in the positive control cell line HCC 1806 (Figure 6A). Patient to patient variation precluded statistical significance for most comparisons, but significantly less IL-8 was detected in all ER^neg^ BCSCs compared to ER^neg^ whole tumor samples. The IL-8 protein data was in agreement with the mRNA data in that there was great variation in levels of expression within the patient population and that, on average, cells from ER^neg^ tumors had more IL-8 than cells from ER^pos^ tumors.

## Discussion

The findings that breast cancer tumors contain a subpopulation of cells that are not effectively targeted by chemotherapeutic agents and radiation has led to cellular and molecular analyses of benign and cancerous breast tissues [32–34]. In this study we compared the gene and protein expression of ERα and ERβ, PR, IL-6 and IL-8 in cells isolated from invasive ductal carcinomas and benign breast tissue specimens. These data reveal variable levels of hormone receptors and cytokine expression which may explain the inconsistent response of breast cancers to hormone therapies and suggest a mechanism by which some patients experience recurrent disease whereas others achieve long term remission.

The identification and classification of stem and progenitor cell lineages in breast cancer remains under development. Al-Hajj and colleagues focused on cells with the profile CD44^+^/CD24^−^, Wicha and colleagues added in the ALDH marker, and Clarke and colleagues isolated cells based on p21^CIP1^ and Msi-1 expression [3, 35, 36]. In this study we separated cells by CD49f/CD24 expression and measured CD44 expression ([37] and Figure 2). We found CD49f^+^CD24^+^ cells to be primarily CD44^+^, while all other populations exhibited a range of CD44 expression. Despite variation in stem cell isolation strategies, studies from multiple laboratories report that BCSCs express very little ERA compared to the tumor of origin or to breast cancer cell lines [9, 10, 38, 39]. Data presented here are innovative and expand the field in that we measured gene and protein expression of ERα and ERβ, PR IL-6 and IL-8 in uncultured CD49f/CD24 BCSCs from individual human invasive ductal carcinomas.

A limitation of this study is that we were unable to study BCSC gene and protein expression in the same tumor sample. The rarity of BCSCs and a tumor specimen size on average of 0.2 mg precluded the study of gene and protein expression in the same patients. The average number of cells collected for lin^neg^ FACS populations CD49f^+^CD24^+^ (PP) and CD49f^−^CD24^+^ (MP) was 40,098 and 30,491, respectively. The PP and MP populations were below 50,000 cells on average which required that all FACS cells were used for PLA. Interestingly, in a cell dilution analysis of the sample 102 T we were able to detect IL-8 protein in as few as 5 sorted cells. While this demonstrates the potential sensitivity of this assay, this was not the norm for any other protein. Thus, we could not directly correlate mRNA and protein expression between the same samples, rather gene and protein expression comparisons were made by averaging the results of study populations. We could, however, compare gene and protein expression between BCSCs and their tumor of origin, as well as SCs with their benign tissues of origin.

With the technical limitations in mind, the analysis of these data led to several important conclusions. The variability of gene and protein expression observed in this study reinforces that breast cancers are biologically complex. When data is presented in averages patient-to-patient variability is masked. As we approach the age of personalized cancer care, identifying significant differences between breast cancers will facilitate superior targeted treatment.

Gene expression averages demonstrated low levels of ERA and ERB in BCSCs (Figure 3, bars), while individual data points reveal the range of expression observed between patients (Figure 3, symbols). We detected the highest levels of ERA in ER^pos^ tumors and CD49f^−^CD24^−^(MM) cells; ERB was more variable in these populations. Similar to gene expression, ERα protein expression is also varied in sorted cells from ER^pos^ tumors. Interestingly, ERα protein expression in the CD49f^−^CD24^−^(MM) population is lower than that detected in the other BCSC populations. Gene and protein expression studies were not conducted in the same cells but the high levels of ERA gene expression and low levels of ERα protein expression in the CD49f^−^CD24^−^(MM) population suggest that ERα protein expression in BCSCs may be subject to post-transcriptional regulation as has been demonstrated in cell lines [40–42].

ER^neg^ tumors also exhibited variable ERA and ERB expression, but little ERα protein was detected. Low levels of ERβ protein were detected in ER^neg^ tumors, but it is hard to determine the relevance of this finding, as we could not detect ERβ in reportedly positive cell lines. Some studies report detection of basal levels of ERβ protein in MCF7 and T47D cell lines while others state that ERβ is only present in MCF7 and T47D upon induction [29–31]. We were not able to detect ERβ in MCF7 or T47D by PLA, but in each of these studies, including ours, different antibodies were used.

We cannot yet correlate differences in BCSC gene and protein expression with clinical outcome, as we lack long term patient follow-up. However, given what is known regarding IHC ER staining of breast cancers and treatment response, we suggest that there will be a correlation of patient outcome with BCSC ER status. Our data suggest that BCSCs with PLA values above 2.5 are likely ER^pos^. They may therefore respond to hormonal treatments in a similar fashion as breast cancer receiving an ER^pos^ IHC evaluation by ASCO guidelines [14]. Examination of BCSC ER by PLA reveals that most ER^pos^ cancers contain BCSCs that do not express ER protein. Individual data points reveal that there is a range of expression between tumors, but that ER expression is negligible in most BCSCs (Figure 6C). The discrepancy of hormone status between various BCSCs and the tumor may have serious therapeutic implications. In theory, treatments targeted to ER^pos^ cells would not affect the ER^neg^ BCSCs in the tumor. This may result in ineffective eradication of BCSCs and the means for tumor recurrence. The patients in this study will be followed to determine the clinical outcomes associated with variable BCSC hormone receptor expression.

In general, PR expression was low in BCSCs and lower than that in tumors and benign tissues. Again, there was variability in expression between patients as well as in the correlation of ER to PR expression. Interestingly, when hormone receptors were compared between BCSC and SC, only PR was significantly higher in expression. The difference in PR protein expression in BCSCs from ER^pos^ tumors versus ER^neg^ tumors was not statistically different. We found no correlation between ERα and PR protein expression. It may be that while expressed, the level of ER activity varies between breast cancers or other untested co-activators such as HER4 are low [25]. We cannot comment further on this, as we did not study the activity of ER or expression of HER4. However, these data agree with other expression studies in mice and humans which also report low levels of PR expression in CD44^high^/CD24^high^ sorted cells [9, 10].

IL-6 is found in ER positive tumors and is thought to synergize with estrogen to increase ER transcriptional activity [19, 21], while IL-8 is inversely correlated with ER expression [23]. IL-6 has been implicated in maintaining a feedback loop between cancer stem cells and non-stem cancer cells through induction of epithelial-mesenchymal transition [43], and IL-8 has been implicated in BCSC self-renewal [39]. Thus, increased IL-6 and decreased IL-8 could indicate better responses to tamoxifen or aromatase inhibitors. In this study IL-6 expression was detected in both ER^pos^ and ER^neg^ tumors. Contrary to other studies [21], IL-6 expression was slightly higher in ER^neg^ tumors. In ER^pos^ tumors, the CD49f^−^CD24^−^(MM) population exhibited the highest gene expression but the lowest protein detection suggesting post-transcriptional regulation of IL-6 in BCSCs as demonstrated in HeLa cells [44]. IL-6 protein was highest in benign tissue and CD24^neg^ cells. Studies have shown that ER^pos^ tumors were responsive to IL-6 therapy due to low autocrine levels of IL-6, while ER^neg^ tumors were not responsive potentially due to high autocrine levels of IL-6 [45]. Thus, higher levels of IL-6 found in benign SCs may protect them from unwanted side effects of IL-6 therapy. However, similar to ER PR data, the IL-6 data suggest that tamoxifen and aromatase inhibitors would likely target the largest tumor BCSC population represented by CD49f^−^CD24^−^(MM), but not the scarce stem/progenitor populations represented by CD49f^+^CD24^+^(PP), CD49f^+^CD24^−^(PM) or CD49f^−^CD24^+^(MP) cells.

IL-8 gene and protein expression were highly variable in benign tissue and tumors, and in both benign SC and BCSC populations. IL-8 levels were consistent with an inverse correlation between IL-8 expression and ER tumor status. IL-8 levels were higher in benign SC and BCSC populations than benign tissue or whole tumor and highest in the CD49f^−^CD24^−^(MM) population. IL-8 protein was lower in benign SCs and BCSCs than in tissue/tumor of origin, but significant levels were still detected. IL-8 has been implicated in regulating the epithelial-mesenchymal transition [46], and blocking IL-8 signaling selectively depletes ADLH^+^ stem cells [47]. Thus targeting IL-8 positive BCSCs may benefit patients with high IL-8 levels. In addition the inverse correlation between IL-8 and ERα expression could provide a level of diagnostic confirmation.

## Conclusions

Estrogen and progesterone receptors and cytokines IL-6 and IL8 gene and protein expression in tumors and BCSCs among patients was highly variable. In addition, the data presented here indicate that the gene and protein expression of BCSCs may vary from that of other cells within a tumor. Because BCSCs are a rare population of cells within a tumor, they are not accurately tested by random sampling of whole tumor specimens [48]. Thus, from a clinical perspective, determining the gene and protein status of directly isolated BCSCs from each patient tumor may prove to be critical for informed care management.

## Electronic supplementary material

Additional file 1: Figure S1: ERβ protein PCR results. A) Protein PCR for detection of ERβ in MCF7, T47D and HCC1806. Probe set one: S2015, polyclonal antibody divided and labeled with oligo A or oligo B (Epitomics, Burlingame, CA). Probe set two: Millipore 05–824 monoclonal Ab labeled with oligo A (Millipore, Billerica, MA) and NB100-92457 monoclonal Ab labeled with oligo B (Novus Biological, Littleton, Co). B) Protein PCR results for benign tissue and SCs and tumor and BCSCs using probe set 1. (TIFF 64 KB)

## Abbreviations

BCSC: Breast cancer stem/progenitor cells
Benign SC: Benign breast stem/progenitor cells
ERA: Estrogen receptor alpha gene expression
ERα: Estrogen receptor alpha protein expression
ERB: Estrogen receptor beta gene expression
ERβ: Estrogen receptor beta protein expression
ER^pos^: ER positive tumor by IHC
ER^neg^: ER negative tumor by IHC
IHC: Immunohistochemistry
PR: Progesterone receptor gene expression
PR: Progesterone receptor protein expression
IL-6: Interleukin-6 gene expression
IL-6: Interleukin-6 protein expression
IL-8: Interleukin-8 gene expression
IL-8: Interleukin-8 protein expression.
